# A Combined Model and Data-Driven Approach for the Determination of Rotor Temperature in an Induction Machine

**DOI:** 10.3390/s21134512

**Published:** 2021-06-30

**Authors:** Razvan Mocanu, Alexandru Onea, Constantin Catalin Dosoftei

**Affiliations:** Department of Automatic Control and Applied Informatics, Gheorghe Asachi Technical University of Iasi, Bulevardul Profesor Dimitrie Mangeron 67, 700050 Iași, Romania; aonea@ac.tuiasi.ro (A.O.); cdosoftei@ac.tuiasi.ro (C.C.D.)

**Keywords:** AC machines, neural network applications, recursive estimation, fault diagnosis

## Abstract

The need for protection of electrical machines comes as a demand of safety regulations in the automotive industry as well as a result of the general desire to obtain a robust and reliable electric powertrain. This paper introduces a hybrid method for estimating the temperature of the rotor of an Induction Machine (IM) based on a Nonlinear Autoregressive Network with Exogenous inputs (NARX) used as a prediction function within a particle filter. The temperature of the stator case is measured, and the information is used as an input to a NARX network and as a variable to a thermal process with first-order dynamics which serves as an observation function. Uncertainties of the NARX and thermal model are determined and used to correct the posterior estimate. Experimental data are used from a real IM test-bench and the results prove the applicability and good performance.

## 1. Introduction

The topic discussed in this paper is contextualized in the field of electric machine protection. The goal is to protect the rotor of the Induction Machine (IM) from overheating to increase the lifetime of the electrical machine. Improper thermal protection can lead to serious defects and even life-threatening situations. The method presented here requires a collection of data representing a sparse range of operating points of the electrical machine (i.e., phase currents, voltages, angular speed, etc.). The RMS-current and the temperature of the stator are measured with onboard sensors along with the angular speed while the rotor current is derived from the current controller. With the a priori data we parametrized the thermal model which serves as an observation function and trained a Nonlinear Autoregressive Network with Exogenous inputs (NARX) neural network to predict state transitions of the temperature. We tested the algorithm with input data acquired from a Belt-Driven Booster (BDB) test-bench for 48 V Mild-hybrid Electrical Vehicles (Mild-HEVs).

To justify the importance of this work we must first contextualize it in the automotive industry. It can be difficult to include in the end product expensive components such as infrared sensors, mainly due to two reasons. First is the extra cost, which is hardly acceptable in a tough competitive industry. Secondly, the reliability and accessibility of the sensors play a key role. For example, often the sensor must be positioned in hard-to-reach locations to provide useful data. This is the case of infrared sensors used in performance electric motors with fast thermal dynamics. Impurities, long-term exposure to mechanical vibrations can affect the precision of the measurements. We meet these rough conditions often in vehicles.

Acquiring information about the temperature of the IM is important because the thermal behavior of the IM influences the performance of the electrical drive train. Electrical machines should be monitored and diagnosed as per safety criteria [1,2,3].

The IM can be exposed to temperatures ranging from $-50{\;}^{\circ }$C to more than $150{\;}^{\circ }$C in Mild-HEVs. Asymmetric power supply caused by inverter faults can lead to thermal overload of the IM. Thermal overload can also be caused by inadequate cooling, air-gap eccentricity, or overstress of the electrical machine (i.e., requesting high power from the IM for an extended time) [4]. Aluminum, copper, or their alloys are primary materials for the rotor bars of IM, while laminated steel is used for the inner portion of the stator. To ensure the protection of the electrical machine, we need to know the melting points and understand the deformation characteristics of the components. If an early failure is not detected, deformation of the rotor bars will worsen, leading to the raising of the bars to where the stator core and stator windings are damaged. Implicitly, the motor performance is altered, and in the worst-case scenario, torque at the shaft becomes erratic. The algorithm points to solve the problem of determining the temperature of the rotor of an IM used in Mild-HEVs without using a rotor temperature sensor. For validation purposes, we acquire the temperature of the rotor through an infrared sensor. Towards this, a hole was drilled into the case of the IM and the infrared sensor was placed with the direct sight of the rotor. On the final product, namely the BDB, this temperature sensor will not be present due to reliability issues mentioned above and to keep the production costs low. The target was to place the infrared sensor as close as possible to the assumed hot-spot considering that in an IM most of the high temperatures are located at the joints of the aluminum/copper bars-end with the end rings. Previously, we implemented a particle filter for estimation of the hot-spot temperature on the stator windings of a Permanent Magnet Synchronous Machine (PMSM) [5]. Different approaches to solve this problem have been investigated. The Nelder-mead method is employed towards nonlinear parameter estimation of a thermal model and the impedance response over a temperature range is determined. The impedance is measured with a specialized instrument (N4L precision impedance/LCR analyzer) and relates to the temperature of the IM [6]. Thermal Impulse Response modeling is employed to forecast the temperature in different areas of an electric machine to localize the hot spots. A heat narrow impulse is applied convolving with the loss of the machine and the temperature is calculated. The method is suitable in the design phase of the electrical machines but is also useful because it provides prior information about the most critical areas [7]. The paper describes an attempt to estimate the spatial distribution of temperature of a permanent magnet in an electrical machine based on the estimation of BEMF harmonics. The authors propose an empirical quadratic law to capture the relation between the maximum and the minimum temperatures across an interior permanent magnet synchronous machine and they emphasize the need for a ground-based approach for the mathematical model [8]. A multiple-scale method is applied to obtain the homogenized heat equation [9]. The case of an automotive switched reluctance machine is considered, and the parameters of a lumped thermal network are identified [10]. In another study, it is pointed out the effect of disturbance of the resolver signal on the temperature of an IM. The authors propose a method of estimation of stator temperature starting from the fixed frame where a DC signal is injected, and the stator resistance is estimated in a field-oriented control scheme [11]. Pulse injection with zero voltage average is used to estimate rotor parameters [12]. Contrary to invasive methods, optimal or sub-optimal filters can be used to estimate the temperature of electrical machines considering the dependence between the magnetic flux and rotor temperature. These approaches require prior knowledge of the electrical subsystem and often assume Gaussian uncertainties [13,14]. A method is described for estimating the temperature of the magnet of a PMSM using the dq frame model within a Kalman filter. The VSI distortion term is included in the estimator [15]. The equivalent circuit parameters of an IM are estimated by a particle swarm optimization algorithm in steady-state conditions. The authors emphasis the applicability of the algorithm for continuous working conditions but mention the lack of precision in transient operations [16]. Estimation of internal resistance of the rotor through model reference adaptive is a technique used widely; however, it requires the knowledge of the machine nominal parameters to provide good results [17]. Additional search coils are mounted inside a reluctance machine on the stator windings to provide a direct measurement of stator flux and further provide an estimation of temperature and shaft position [18]. In open-end wingdings PMSM applications where small torque ripples are acceptable, zero current sequences are used to determine the stator resistance and further the temperature [19]. The time constant of a first-order thermal model is computed based on a polynomial function of the speed and stator temperature of a PMSM [20]. A design procedure for guaranteed state estimation of unmeasurable states with practical application to induction machines is discussed [21]. Related to the approach involving particle filter we mention a few remarks. The particle filter is based on an effective sequential Monte Carlo method to solve the recursive Bayesian filtering problem [22,23]. The computational power allows now to use the particle filter in a wider range of applications. In the automotive world is used to estimate the traffic conditions [24]. Temperature estimation of a satellite is implemented considering a double lumped thermal model [25]. Particle filters are used for fault prediction and diagnosis. IGBTs faults are predicted with application in power electronics [26]. A simulation framework with practical results for temperature estimation of IGBT is detailed in the work of authors [27]. Throughout the mentioned publications in this field we could practically divide the described methods into three categories: (a) methods that rely on additional hardware (e.g., search coils, real-time impedance measurement devices, magnetic flux sensors, etc.). (b) methods that are invasive and can alter the control command of the electrical machine (e.g., DC injection, impulse injection) and methods that rely on a precise thermal model and precise knowledge of nominal parameters.

The method proposed in this paper has the advantage of being simple despite the complicated-look of the particle filter, it is straightforward and provides good results. It is decoupled and can be modularized, allowing an update or change to the prediction and observation model in a ‘plug and play’ manner. It can be seen as three individual parts: two information channels and one merging strategy. Except for the fairly common particle filter, the other two information channels can be adjusted or replaced without any impact on the particle filter implementation, thus the overall implementation is somewhat simple. Furthermore, we mention the most important features:

- The algorithm consumes data that is already available on most standard architectures of power electronics used in electric machine control (i.e., phase currents sensors, stator temperature sensor, position, and speed encoder).
- The method does not require additional components (e.g., real-time precision impedance measurement, search coils, etc.)
- The method is not invasive and does not alter the control commands to the electrical machine.
- In the common NARX network, the state is updated with the actual output of the network. In the proposed algorithm, we update the state of the NARX network with the posterior estimate. The posterior estimate is obtained after merging with the particle filter the two information channels: the NARX and the thermal model. This corrects the prediction of the neural network in the prediction stage. Thus, is obtained a conditioned output of the NARX model with the thermal model which in the end improves the precision of the estimated value. This approach is distinctive, and it was not investigated before to the problem of temperature estimation.

The paper continues with a brief description of the self-contained BDB with IM in Section 2, while Section 3 introduces the mathematical models considered in the particle filter. In Section 4, the equations of the recursive Bayesian filter and particle filter are provided with the application for rotor temperature estimation. In Section 5 are presented the experimental results from the motor-load test-bench. Finally, Section 6 concludes this work and points to some possible improvements.

## 2. System Description—Belt-Driven Booster

**Figure 1:**
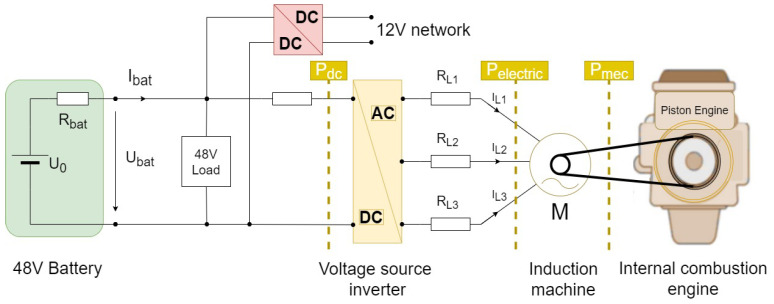
A conceptual illustration of a 48 V electrification system.

The BDB consists of a three-phase IM with an integrated inverter and control unit which is capable of offering a continuous power of 6KW and a torque of 60NM on a 48 V power net system. The system is especially efficient in diesel hybrid cars. The nitrogen oxide emissions are reduced during accelerations from low speed when some of the torque is produced by the BDB. This system allows a fast engine start and energy recuperation. Additionally, engine-off coasting can be permitted (i.e., the internal combustion engine is shut down after the vehicle reaches a higher speed and the propulsion is ensured by the BDB). Additionally, the IM can be maintenance-free mainly, due to the lack of slip rings, thus reducing the costs. The commands to the BDB are sent through a Controller Area Network (CAN)/Flexray channel by the electrical vehicle main controller, thus the system presents itself as a self-contained, compact solution Overall, the 48 V systems (DC/DC, inverter) are, at the moment, one of the most affordable and quick solutions for electrification in the automotive industry. Figure 1 shows a conceptual representation of a 48 V traction system.

**Figure 2:**
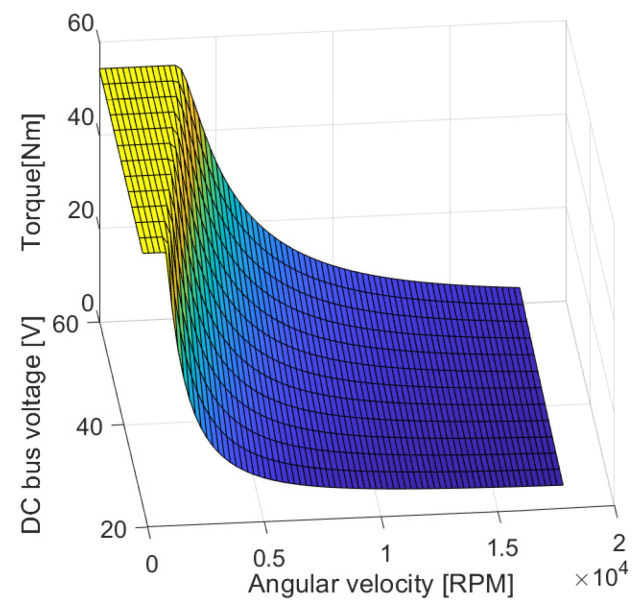
The speed-toque characteristic w.r.t to the DC voltage bus.

The dynamics of the electrical quantities of the IM are modeled by a 4th order system. The variables and parameters are expressed in the two-axis reference frame. The voltage equilibrium equations of the stator ($V_{ds}$, $V_{qs}$) and of the rotor ($V_{dr}$, $V_{qr}$) are defined by (1):

(1) $$\begin{array}{cc} V_{qs} & =R_{s}i_{qs}+\frac{d\psi_{qs}}{dt}+\omega \psi_{ds}\\ V_{ds} & =R_{s}i_{ds}+\frac{d\psi_{ds}}{dt}-\omega \psi_{qs}\\ V_{qr} & =R_{r}i_{qr}+\frac{d\psi_{qr}}{dt}+\left(\omega -\omega_{r}\right)\psi_{dr}\\ V_{dr} & =R_{r}i_{dr}+\frac{d\psi_{dr}}{dt}-\left(\omega -\omega_{r}\right)\psi_{qr} \end{array}$$

where ω is the mechanical angular velocity, $\omega_{r}$ is the electrical angular velocity, $i_{ds}$, $i_{qs}$ are the stator currents and $i_{dr}$, $i_{qr}$ are the rotor currents. The rotor is squirrel-cage type and $V_{qr}=0$, $V_{dr}=0$. $R_{s}$ and $R_{r}$ are the stator and rotor resistances, respectively. The stator fluxes $\psi_{qs}$, $\psi_{ds}$ and the rotor fluxes $\psi_{qr}$, $\psi_{dr}$ are defined by:

(2) $$\begin{array}{cc} \psi_{qs} & =L_{s}i_{qs}+L_{m}i_{qr}\\ \psi_{ds} & =L_{s}i_{ds}+L_{m}i_{dr}\\ \psi_{qr} & =L_{r}i_{qr}+L_{m}i_{qs}\\ \psi_{dr} & =L_{r}i_{dr}+L_{m}i_{ds} \end{array}$$

where $L_{s}=L_{ls}+L_{m}$, $L_{r}=L_{lr}+L_{m}$ define the total inductance of the stator and of the rotor, respectively. $L_{m}$ is the mutual inductance and $L_{ls}$ and $L_{lr}$ are the leakage inductance of the stator and rotor, respectively. $i_{r}$ is an estimated value of the magnetization current. In our case $i_{r}$ was approximated by low-pass filtering $i_{ds}$ with the time constant of the rotor electrical dynamics $\tau_{r}=L_{r}/R_{r}$. The speed-torque characteristics is plotted concerning the DC voltage bus in Figure 2. The parameters of the IM are listed in Table 1.

$f_{s}$ is the inverter switching frequency, η denotes the angular speed, *V* is the nominal DC voltage bus, $P_{n}$ is the nominal mechanical power. Index *n* is used for nominal values.

## 3. The Mathematical Models of the State Transition and Observation Functions

We propose to use the model defined by (3) considering that the major heat source in an IM is the rotor and the stator will follow to a certain extent the rotor temperature curve. The model is a simple heat transfer function of first-order which includes a thermal resistance through which heat is dissipated and a thermal capacitance that absorbs heat. τ is a time constant introduced for realizability. The model is not meant to be a precise description of the thermal process but rather to act as a filter/preconditioner of the NARX network.

(3) $$\tau \frac{dT_{\alpha }\left(t\right)}{dt}+T_{\alpha }\left(t\right)=\alpha_{1}\frac{dT_{s}\left(t\right)}{dt}+\alpha_{2}T_{s}\left(t\right)$$

$T_{\alpha }$ is the rotor temperature computed from the model (3) and $T_{s}$ is the measured stator temperature. The coefficients $\alpha \in R^{2x1}$ are calculated offline with the least-squares equation:

(4) $$\alpha ={\left(C_{\alpha }^{*T}C_{\alpha }^{*}\right)}^{-1}C_{\alpha }^{*T}T_{r}^{*}$$

where $C_{\alpha }^{*}\in R^{Nx2}$ is defined as:

(5) $$C_{\alpha }^{*}=\left[\Delta T_{s}^{*}\;\;T_{s}^{*}\right]$$

and $\Delta T_{s}^{*}$ is the gradient of the stator temperature sensor:

(6) $$\Delta T_{s}^{*}=\left[\begin{array}{c} (T_{s}^{*}\left[1\right]-T_{s}^{*}\left[0\right])/h\\ \ldots \\ (T_{s}^{*}\left[N\right]-T_{s}^{*}\left[N-1\right])/h \end{array}\right]$$

τ is the time constant of the low-pass filter introduced for realizability and *h* is the sampling time of the algorithm.

The NARX is a recurrent network with feedback connections encircling several layers of the network. The NARX structure is built on the linear ARX model, which is generally used in time-series modeling [28]. We use the structure of the recursive neural network with delay action because it accounts for the intrinsic memory of thermal processes.

The defining equation for the NARX model is:

(7) $$T_{net}\left[k\right]=narx\left(i_{r}\left[k\right],i_{s}\left[k\right],\eta \left[k\right],T_{s}\left[k\right],T_{net}\left[k-1\right]\right)$$

where $i_{s}$ the stator current and η is the angular speed. We added one hidden layer, fully connected, to the network structure which is enough to capture the dynamics of the predicted temperature $T_{net}$. Increasing the size of the hidden layer or the number of layers did not contribute to significant improvements in the precision but rather to the increase of the computational run-time. Thus, $f_{1}$ is the hidden layer’s sigmoid activation function, while $f_{2}$ is the output layer’s linear activation function. The hidden layer’s weights and biases are $W_{li,j}$ and $b_{Li}$, whereas the output layer’s weights and biases are $W_{Li}$ and $b_{0}$. The indexes are in the range of *i* = 1…10 and *j* = 1…4.

### Data Acquisition and Network Training

**Figure 3:**
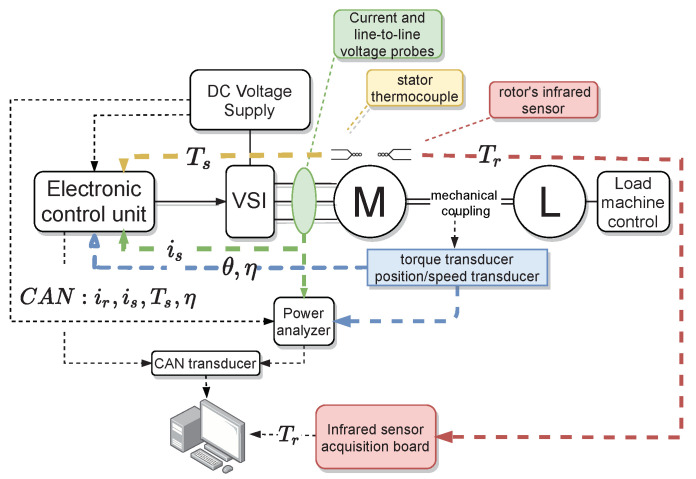
Illustration of the data acquisition chain.

**Figure 4:**
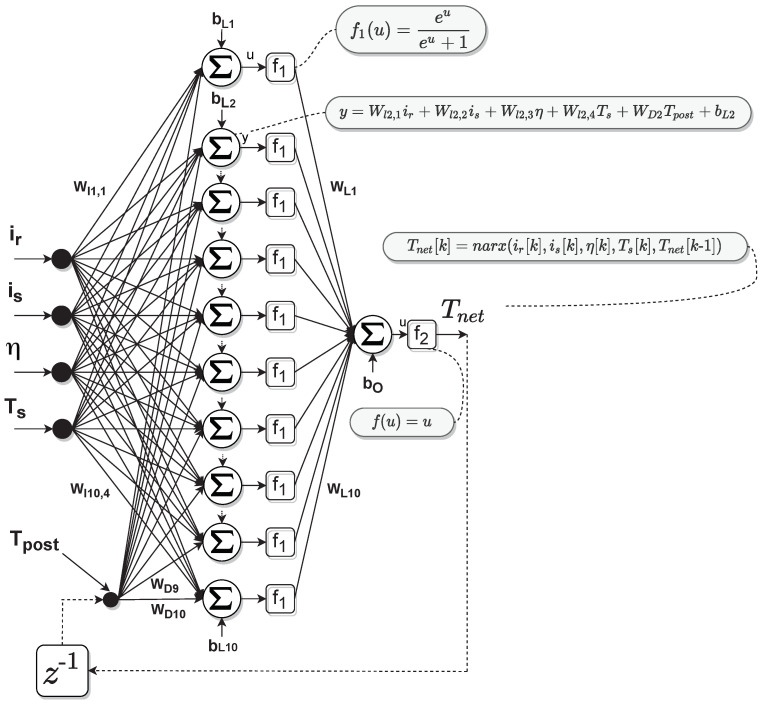
NARX Neural Network.

Figure 3 illustrates the data acquisition chain. The data available at the level of the Electronic Control Unit (ECU) is the RMS stator current ($i_{s}$) acquired through the phase current sensors, the stator temperature $T_{s}$ acquired through the stator thermocouple, the mechanical position of the shaft acquired through the position sensor. The ECU estimates the rotor temperature and calculates the angular speed from the position sensor. The role of the power analyzer is to provide analysis of the active and reactive power and can provide a redundant measurement of the phase currents, voltages, and torque. In the training process, we used data received from the ECU through the CAN bus transducer ($i_{r}$, $i_{s}$, $T_{s}$, η) and the real rotor temperature from the infrared-sensor acquisition board. The currents are acquired with a sampling time of 100μs, the angular speed is acquired with 10 ms, and the temperature with a sampling time of 100 ms. Because of the different sampling times, we performed linear interpolation for the low-sampling variables to have the same size of data before the training process. Figure 4 illustrates the structure of the neural network.

For training the network we recorded different operating points with ranging speeds from 0 to 15,000 RPM and torque from 0 to 50 Nm summing a total of approximately 5 h of recordings.

**Figure 5:**
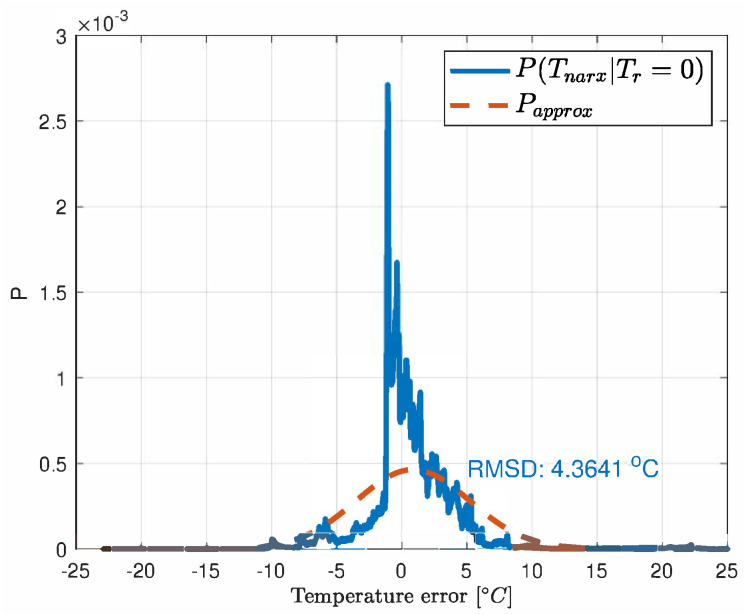
The error distribution of the neural network.

The model was validated on the test set by comparing the estimated temperature ${\hat{T}}_{net}$ with the real temperature acquired with the infrared rotor temperature sensor $T_{r}$ Figure 5 shows the error distribution of the NARX network.

The training was carried out using Matlab’s neural network utility (trainlm) and included two steps of preparation: first, converting data from DAT representation (test-bench data acquired via XCP protocol) to MAT format (Matlab’s particular format), and second, normalizing all variables prior to the training process. The weights $W_{li,j}$, $W_{Li}$ and biases $b_{Li}$, $b_{0}$ are updated during the backpropagation training with the Levenberg-Marquardt (LM) optimization algorithm. For training, we used the open-loop architecture in which the real temperature of the rotor is used as feedback in the backpropagation algorithm instead of the estimated temperature. This offers two benefits. The first is that the feedforward network’s input is more accurate. The second benefit is that the resulting network has a feedforward design, allowing for the usage of a more efficient training procedure such as the LM method. Comprehensive literature and one of the earliest applications of the LM method to neural network training is documented by the authors of [29,30]. This approach is still one of the fastest for training intermediate-size feedforward neural networks of up to several hundred weights [30]. The implementation of the LM algorithm is fairly common, and a Matlab-specific example may be consulted here [31].

**Figure 6:**
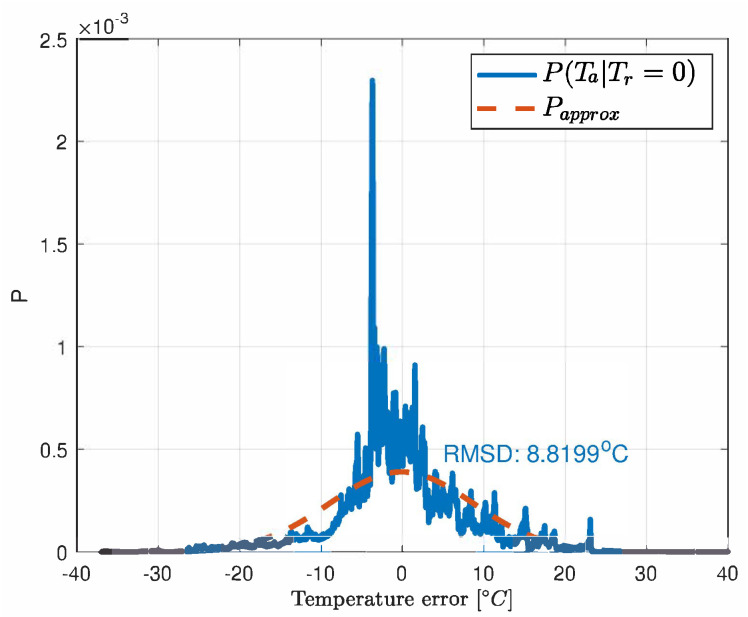
The PDF of the observation model.

The error Probability Density Function (PDF) of the neural network and observation model as derived from the test-bench data are depicted in Figure 5 and Figure 6.

## 4. Particle Filter for Rotor Temperature Estimation

**Figure 7:**
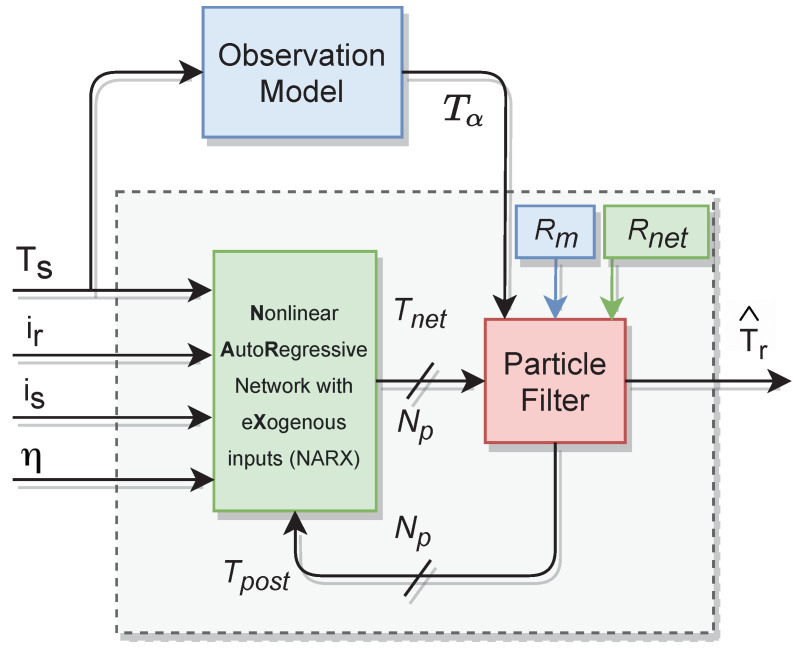
Block representation of the estimator.

Figure 7 is a block representation of the estimation strategy. First, the inputs stator temperature $T_{s}$, rotor current $i_{r}$, stator current $i_{s}$, angular speed η and the previous posterior temperature set $T_{post}$ feed the recursive neural network block and several $N_{p}$ particles are obtained. The PF block pulls a measurement out of the observation model and merges the uncertainties PDF (i.e., Gaussian distributed $R_{m}$ and $R_{net}$) around the newly estimated temperature.

The motivation of using the particle filter for the task of merging data from models (3) and (7) is that the predicted temperature with the NARX network is nonlinear and both models can reveal non-Gaussian noise. In our thermal process the uncertainties can be roughly approximated with the Gaussian distribution (Figure 5 and Figure 6). However, this can vary with the positioning and precision of the sensors. Except for the few mentioned papers, the particle filter was not used in the temperature estimation but it can undertake this task well since the heat exchange is a slow process and computational power is enough for this task. In the prediction step of the recursive Bayesian filter, the temperature $T_{net}$ is calculated as in (7). The corresponding PDF of the prior is given by the Chapman-Kolmogorov equation [22,23] adapted to our set of data *d*:

(8) $$\begin{array}{cc} \underset{\mathrm{prior}\;\mathrm{PDF}}{\underset{︸}{p\left(T_{net_{k}}\;/\;d_{1:k-1}\right)}}=\int & \underset{\mathrm{transition}\;\mathrm{model}\;(\mathrm{NARX}\;\mathrm{net}\;\mathrm{function})}{\underset{︸}{\begin{array}{c} p(T_{net_{k}}\;/\;T_{net_{k-1}},T_{s_{k-1}},\eta_{k-1},..\\ ..i_{r_{k-1}},i_{s_{k-1}}) \end{array}}}\;\\ \cdot & \underset{\mathrm{posterior}\;\mathrm{PDF}}{\underset{︸}{p\left(T_{net,k-1}\;/\;d_{1:k-1}\right)}}dT_{net_{k-1}} \end{array}$$

where *p* denotes the probability, *d* defines the set of data (will be define below) and *k* is the iteration index. Thus, to calculate the prior rotor temperature the NARX net is sampled (i.e., Equation (7) is evaluated) and the weighting is done according to the prediction of the observation model (3). Consequently, the prior precision depends on the precision of the NARX prediction and posterior precision. Following, the posterior PDF is derived from the prior density function of the neural network (8) and from the observation function (3) [32,33] with a normalization factor:

(9) $$\begin{array}{c} \underset{\mathrm{posterior}\;\mathrm{PDF}}{\underset{︸}{p\left(T_{net_{k}}\;/\;d_{1:k}\;\right)}}=\underset{\mathrm{observation}\;\mathrm{model}}{\underset{︸}{p\left(T_{\alpha_{k}}\;/\;T_{net_{k}}\right)}}\;\underset{\mathrm{prior}\;\mathrm{PDF}}{\underset{︸}{p\left(T_{net_{k}}\;/\;d_{1:k-1}\right)}}\\ \underset{\mathrm{normalization}\;\mathrm{PDF}}{\underset{︸}{/p\left(T_{\alpha_{k}}\;/\;d_{1:k-1}\right)}} \end{array}$$

The normalization PDF is defined as [22]:

(10) $$p\left(T_{\alpha_{k}}\;/\;d_{1:k-1}\right)=\int p\left(T_{\alpha_{k}}\;/\;T_{net_{k}}\right)p\left(T_{net_{k}}\;/\;d_{1:k-1}\right)$$

In the previous equations $d_{1:k}$ and $d_{1:k-1}$ define the set of data (i.e., observation model predicted temperature $T_{\alpha }$, stator temperature $T_{s}$, angular speed η, rotor current $i_{r}$ and stator current $i_{s}$) available at recurrence *k* and k−1, respectively:

(11) $$\begin{array}{cc} d_{1:k} & =T_{\alpha_{1:k}},T_{s_{1:k}},\eta_{1:k},i_{r_{1:k}},i_{s_{1:k}}\\ d_{1:k-1} & =T_{\alpha_{1:k-1}},T_{s_{1:k-1}},\eta_{1:k-1},i_{r_{1:k-1}},i_{s_{1:k-1}} \end{array}$$

The PF approximates the PDFs with a finite number of samples, rather than computing (10) at each sample time which can be time consuming in the case of complex analytical forms. As a result, a series of samples (particles) characterizes the PDF of the posterior temperature $p(T_{net_{k}}\;/\;d_{1:k})$. An approximation of the functional form of the posterior PDF is obtained for an infinitely large number of particles. The posterior PDF in its discrete form is defined by [32]:

(12) $$p\left(T_{net_{k}}\;/\;d_{1:k}\right)\approx \sum_{i=1}^{N_{p}}w_{k}^{\left(i\right)}\delta \left(T_{net_{k}}-T_{net_{k}}^{\left(i\right)}\right)$$

The PF generates a set of rotor temperatures and their corresponding weights $\left\{\left(w_{k}^{\left(i\right)},T_{net_{k}}^{\left(i\right)}\right):i=1\ldots N_{p}\right\}$. $N_{p}$ is the number of temperature points in the set. In this work $N_{p}=60$ was determined experimentally and provides satisfactory results. The weights are chosen at each sample time according to the principle of ‘importance sampling’. Hence, the prior PDF is represented as [32]:

(13) $$\underset{\mathrm{prior}\;\mathrm{PDF}}{\underset{︸}{p\left(T_{net_{k}}/d_{1:k-1}\right)}}\approx \sum_{i=1}^{N_{p}}w_{k-1}^{\left(i\right)}\;\underset{\mathrm{transition}\;\mathrm{model}\;(\mathrm{NARX}\;\mathrm{net}\;\mathrm{function})}{\underset{︸}{p\left(T_{net_{k}}\;/\;T_{net_{k-1}}^{\left(i\right)},T_{s_{k}},\eta_{k},i_{r_{k}},i_{s_{k}}\right)}}$$

The PDF of the posterior temperature is given by [22,34]:

(14) $$\underset{\mathrm{posterior}\;\mathrm{PDF}}{\underset{︸}{p\left(T_{net_{k}}\;/d_{1:k}\right)}}\approx \psi \underset{\mathrm{observation}\;\mathrm{model}}{\underset{︸}{p\left(T_{\alpha_{k}}\;/T_{net_{k}}\right)}}\underset{\mathrm{prior}\;\mathrm{PDF}}{\underset{︸}{p\left(T_{net_{k}}/d_{1:k-1}\right)}}$$

In (14) ψ is the normalization constant analogous to (9).

A three-step process is followed to implement the PF: predict, update, re-sample. The NARX function (7) with inputs $T_{s}$, $i_{r}$, $i_{s}$ and η is evaluated in the prediction step for each temperature point from the set of posterior estimates. There is a total of $N_{p}$ temperature points in the posterior set. The obtained vales at the output of the network represent the projected rotor temperatures which are distributed conforming to the prior PDF (13) [22,34].

(15) $$T_{net_{k}}^{\left(i\right)}∼p\left(T_{net_{k}}/d_{1:k-1}\right)$$

Following, the observation model (3) is evaluated in the update step and a set of $N_{p}$ rotor temperature points are weighted based on the PDF of the observation function. For each projected temperature, the weight is determined as follows [22]:

(16) $$w_{k}^{\left(i\right)}=p\left(T_{\alpha_{k}}\;/\;T_{net_{k}}\right)$$

The weights $w_{k}^{\left(i\right)}$ approximate the densities of the rotor temperature points and are normalized so that:

(17) $$\sum_{i=1}^{N_{p}}w_{k}^{\left(i\right)}=1$$

Consequently, the $N_{p}$ particles will be dispersed conforming to:

(18) $$T_{net_{k}}^{\left(i\right)}∼p\left(T_{net_{k}}/d_{1:k}\right)$$

The effect of degeneracy is avoided in the re-sample step where a new group of $N_{p}$ particles are generated from the posterior PDF (14). In this step we integrate (i.e., sum) the values in the set *w* for each particle and we obtain a cumulative real sum denoted in Algorithm 1 as csw∈[0,1]. In the continuous-time domain, this is analog to the cumulative distribution function. We generate a set *X* of $N_{p}$ real values distributed uniformly X∈[0,1]. We used a built-in function that will not be covered in this paper. However, a good reference for uniformly distributed numbers generator can be found here [35]. In the next step, we searched the indices of *X* for each particle that correspond to the csw. These represent the re-sampled indices of the prior estimate $T_{net}$. Following, the posterior estimation of the rotor temperature is determined as the mean value of new posterior particles:

(19) $${\hat{T}}_{r_{k}}=\frac{1}{N_{p}}\sum_{i=1}^{N_{p}}T_{net_{k}}^{\left(i\right)}$$

The re-sampling part of the algorithm is the most computational expensive from the particle filter since it requires the generation of random variables from given distributions. It is more expensive than inferring the neural network. The computational complexity of the re-sampling stage is linear increase with the number of particles. This complexity could be decreased if, for example, we would use a hardware generator of uniform distributions.

The implementation of the estimation algorithm can be synthesized as shown in Algorithm 1. The algorithm is illustrated for 1 sample time.

The parameters of the observation model and particle filter are listed in Table 2.

**Figure 8:**
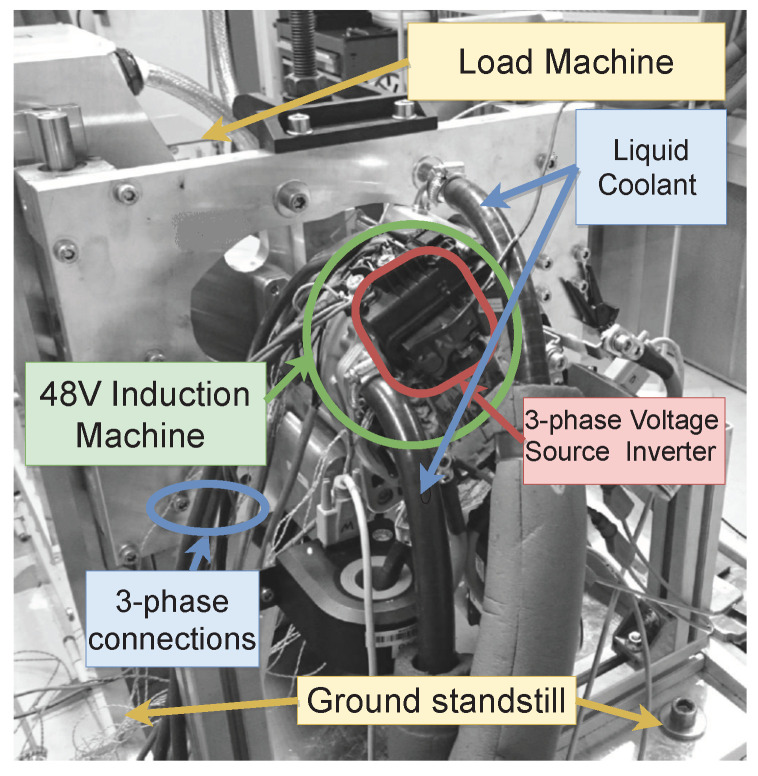
View of the test-bench.

Figure 8 details the test-bench setup.
**Algorithm 1** Particle filter for rotor temperature estimation **Initialization** *Initialize posterior distribution:*
$p\left(T_{net,k-1}\;/\;d_{1:k-1}\right)\leftarrow p_{0}$ *Generate $N_{p}$ particles with initial distribution $p_{0}$ and place them in a set called $T_{post}$.* ***Run*** *Compute* 
$T_{\alpha }\left[k\right]$ $T_{\alpha }\left[k\right]=\frac{h}{\left(h+\tau \right)}\left[\frac{h}{\tau }T_{\alpha }\left[k-1\right]-\frac{\alpha_{1}}{h}T_{s}\left[k-1\right]+\frac{\left(\alpha_{1}+h\alpha_{2}\right)}{h}T_{s}\left[k\right]\right]$ **while**
$i\leq N_{p}$
**do**  Predict  $T_{net}\left[i\right]=narx\left(i_{r}\left[k\right],i_{s}\left[k\right],\eta \left[k\right],T_{s}\left[k\right],T_{post}\left[i\right]\right)$  Update  *Calculate the weight associated with each projected state*:  $w\left(i\right)=1/\sqrt{2\pi R_{m}}exp\left[-{\left(T_{\alpha }\left[k\right]-T_{net}\left(i\right)\right)}^{2}/\left(2R_{m}\right)\right];$  i = i + 1 **end while** Normalize weights:
$w\left(i\right)=w\left(i\right)\sum_{i=1}^{N_{p}}w\left(i\right)$ Re-sample *Sample the discrete distribution*
***w** to generate $N_{p}$ random samples which will represent the posterior $T_{post}$:* $X=rand(N_{p},1)$; fetch $N_{p}$ values from a uniform distribution X∈[0,1]; **for** j = 1: $N_{p}$ **do**  $csw\left(j\right)=\sum_{L=1}^{j}w\left(L\right)$; Cumulative Sum of W; csw∈[0,1] **end for** **for** j = 1: $N_{p}$ **do**  $i_{p}\left(j\right)=argmin_{i}\left|X\left(i\right)-csw\left(j\right)\right|$; $i_{p}$ will be the index of the posterior after re-sampling **end for** **for** j = 1: $N_{p}$ **do**  $T_{post}\left(j\right)=T_{net}\left[i_{p}\left(j\right)\right]$ **end for** ${\hat{T}}_{r}\left(k\right)=\frac{1}{N_{p}}\sum_{i=1}^{N_{p}}T_{post}\left(i\right)$

## 5. Results and Discussions

To simulate and validate the temperature estimation method, recorded data of rotor current, stator current, and stator temperature along with angular speed information is provided to the estimation algorithm. The IM is set in torque control mode while the load machine is controlled in speed mode. The angular velocity of the load machine is controlled by a dedicated voltage source inverter with own control unit. Torque steps with various amplitudes are requested from the BDB. Different ranges of torque and speed setpoints are measured. Validation is done on separate data sets.

**Figure 9:**
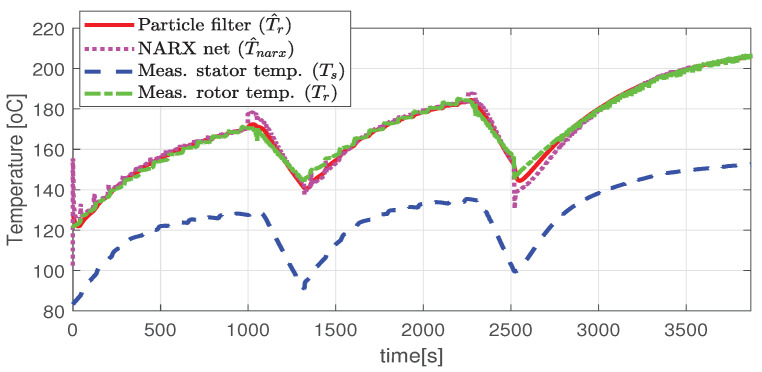
Estimated and actual rotor temperature (test scenario I).

**Figure 10:**
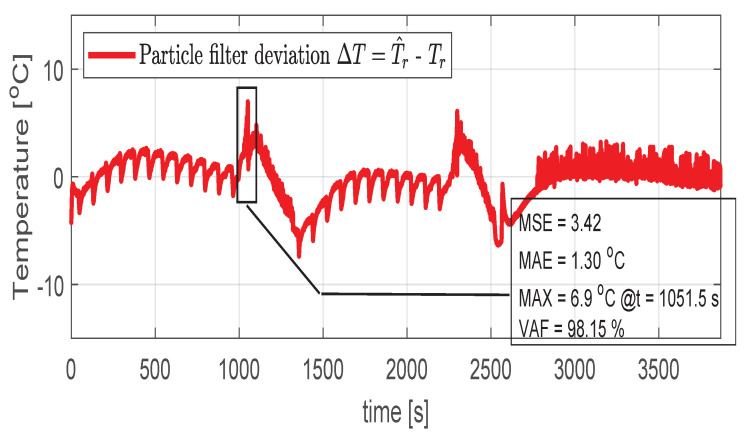
Post estimate temperature deviation (test scenario I).

**Figure 11:**
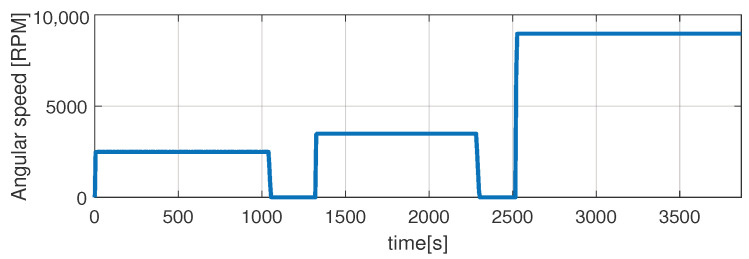
Shaft angular speed (test scenario I).

**Figure 12:**
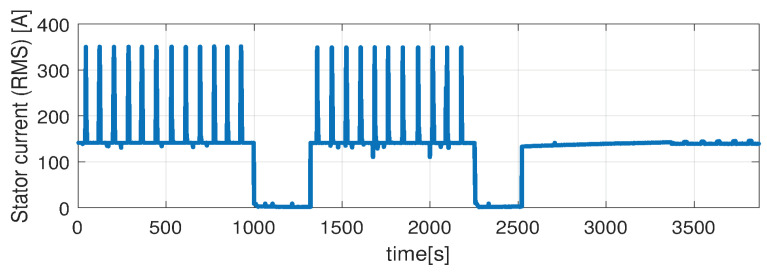
Stator current (RMS) (test scenario I).

**Figure 13:**
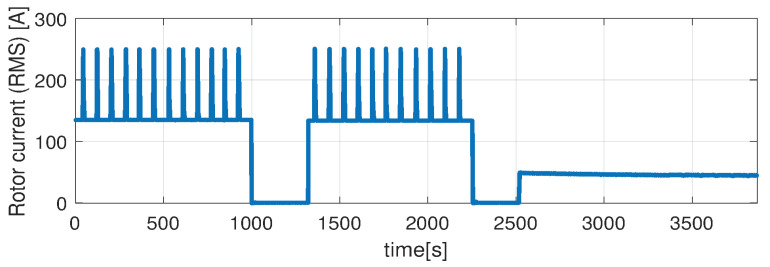
Rotor current (RMS) (test scenario I).

In the first scenario represented in Figure 9, Figure 10, Figure 11, Figure 12 and Figure 13 the IM is accelerated up to 9000 RPM and decelerated to 0 RPM, 0 A to capture part of the cooling behavior. The phase current peaks are up to 350 A and are caused by peak torque requests as can be seen in Figure 12. The magnetization current has a peak of 250 A. The raw (unfiltered) output of the NARX is seen in Figure 9. With the first-order thermal model process the effect is of filtering high frequencies at the output of the NARX network. This is accomplished by the particle filter in the update step where the prior estimated is smoothed considering the uncertainty model of the recursive network. As expected according to the Chapman-Kolmogorov the effect is of filtering ripples in the prior estimate (according to the transition PDF). In all experiments, the initial temperature guess is the stator temperature.

**Figure 14:**
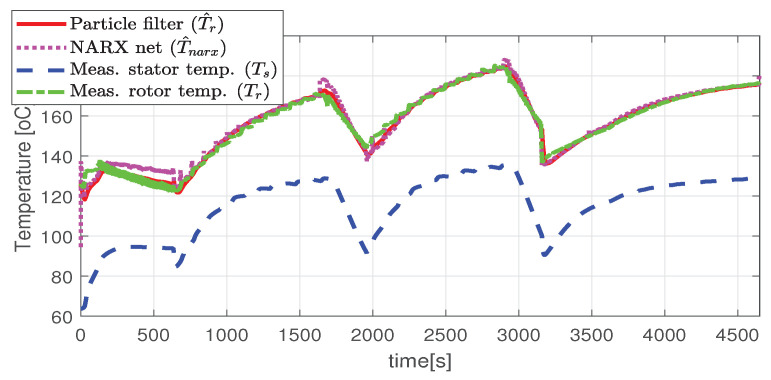
The estimated and the actual rotor temperature (test case II).

**Figure 15:**
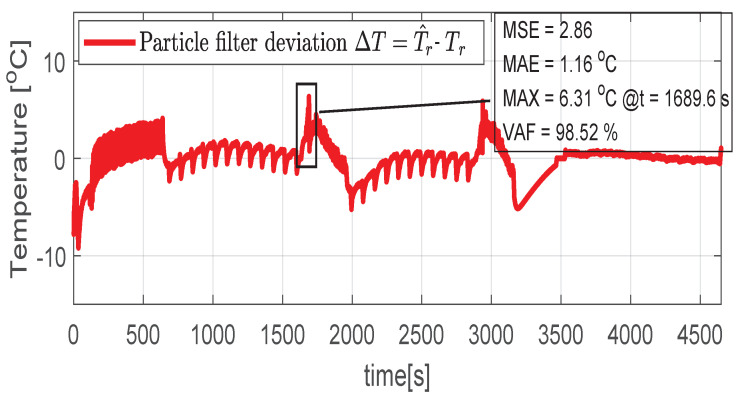
Post estimate temperature deviation (test scenario II).

**Figure 16:**
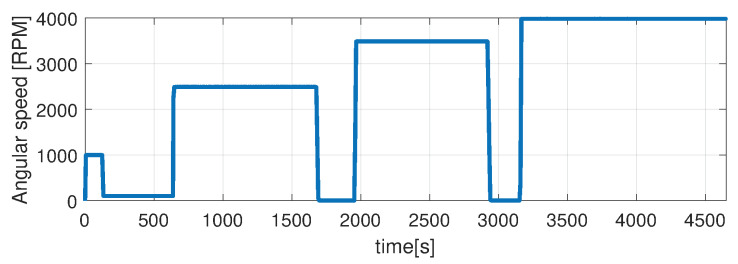
Shaft angular speed (test scenario II).

**Figure 17:**
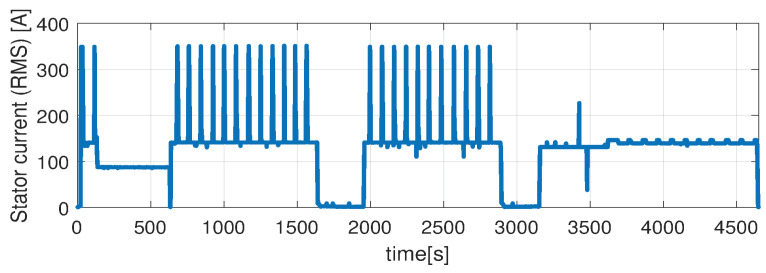
Stator current (RMS) (test scenario II).

**Figure 18:**
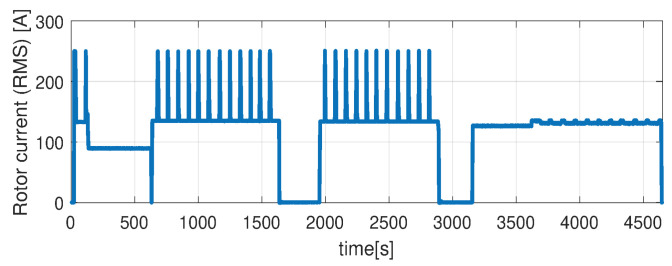
Rotor current (RMS) (test scenario II).

Higher speed interval is tested in the case represented in Figure 14, Figure 15, Figure 16, Figure 17 and Figure 18. The speed is varied in the range of 1000 RPM to 15,000 RPM with different steps. We allow partial cooling of the machine, then we repeat the speed and torque requests. The RMS stator current has a peak of 360 A, while the magnetization current reaches approximately 250 A. Cooling of the machine is captured well by both the thermal model and NARX network and overall we obtain a precision with less than 4 error.

**Figure 19:**
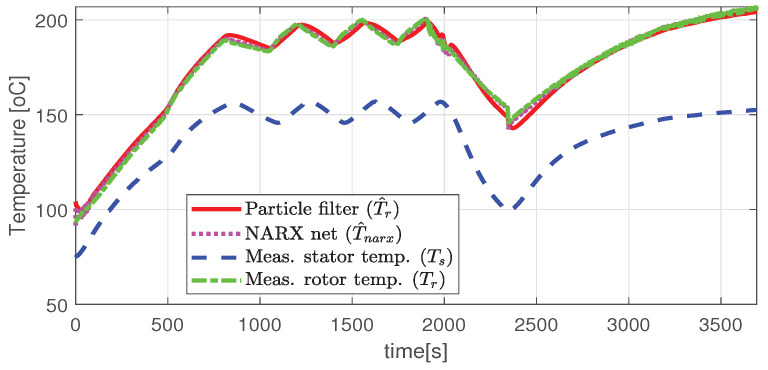
Estimated and actual rotor temperature (test scenario III).

**Figure 20:**
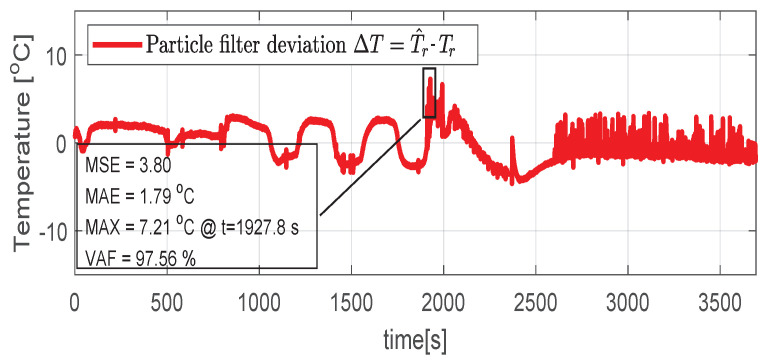
Post estimate temperature deviation (test scenario III).

**Figure 21:**
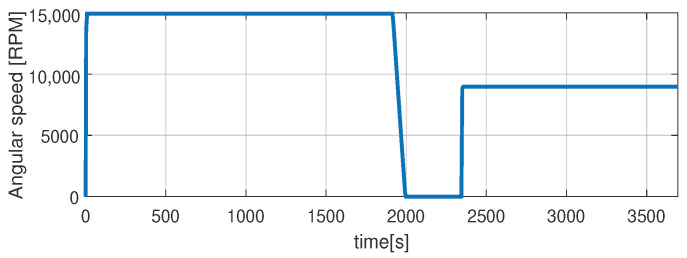
Shaft angular speed (test scenario III).

**Figure 22:**
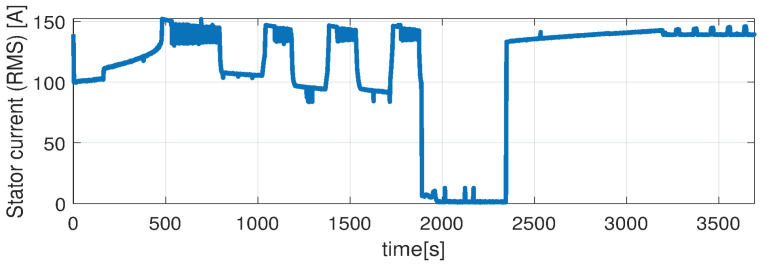
Stator current (RMS) (test scenario III).

**Figure 23:**
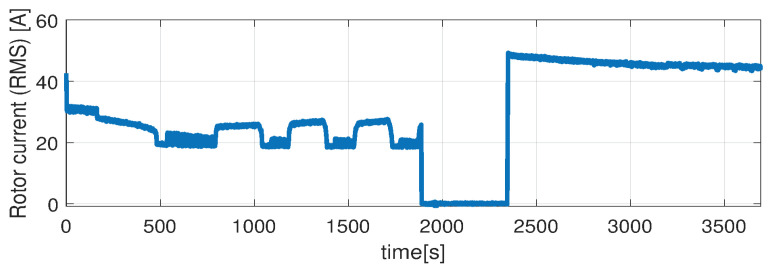
Rotor current (RMS) (test scenario III).

**Figure 24:**
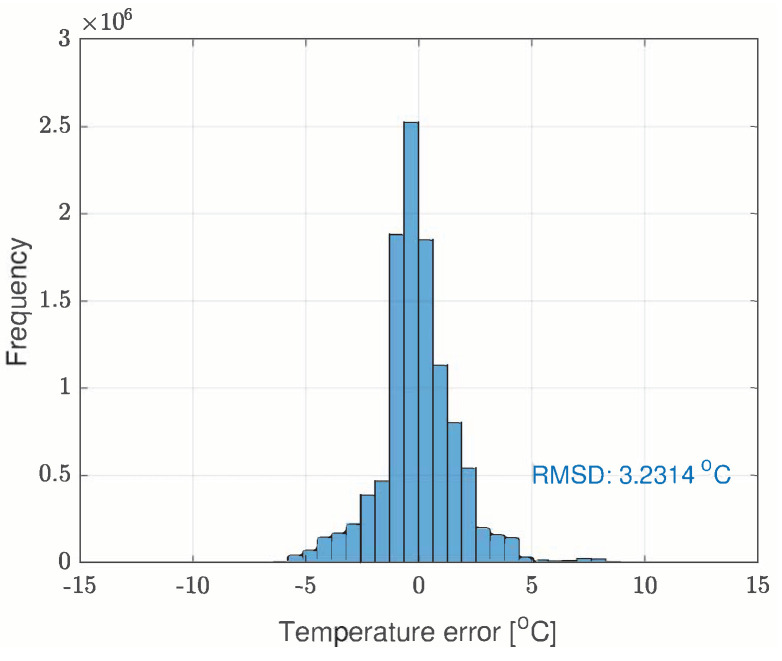
The error histogram of the estimated temperatures over the entire test set.

In the test case represented in Figure 19, Figure 20, Figure 21, Figure 22 and Figure 23 the IM is rotated nearly to the maximum speed. Over the entire test set the estimated temperature shows an average deviation of $3.3{\;}^{\circ }$C (Figure 24).

The performance metrics for the considered test cases are summarized in Table 3.

Figure 24 depicts the error histogram for the entire test set. The parameters of the neural network are provided in Table 4.

## 6. Conclusions

I. There are different development perspectives of this estimation strategy. A recurrent neural network can offer a solution when the thermal modeling capabilities are missing but a high quantity of data are available. Training a neural network involves time for acquiring collections of data. At no supplementary development time, the same data are used to identify the parameters of the observation model and to obtain the uncertainty of the model. Then, we merge both channels through the particle filter. The observation or the transition models can be improved but this means investing more time in the development of a mathematical description with a deeper insight into the thermal physics. The transition and observation functions can be a well-defined mathematical characterization of the thermal process of multiple variables instead of an empirically structured neural network. Thus, one who must decide between these two paths must evaluate the capabilities of the development team and the time constraints. Obtaining a detailed thermal model could involve more time and effort than a simple job of acquiring data on a test-bench, train a network and identify coefficients of a simple first-order process. We saw that a simple first-order process captures well to some extent the cause-effect of the relation *rotor temperature → stator temperature*. Not enough, but this is compensated by the particle filtering with the neural network’s prediction.

II. It is important to highlight that to obtain a precise online estimation of the rotor temperature, the appropriate experiment should be performed built around the targeted application. Therefore, it is advised that the data measurement for identification be done on the actual environment of the IM (e.g., with the BDB mounted inside the vehicle). This is due to possibly different temperature behavior and other heating sources. The external temperature sensor can be placed not only on the stator windings but also on the stator case or at proximity to the electrical machine (such that it exists somewhat thermal conductivity). The sensor could also play the role of an environment temperature sensor and we expect that the NARX network can accommodate during training and can capture the contribution of the external sensor to the estimated temperature of the rotor. We believe that this method is general enough to apply to a wide range of electrical machines and other types of thermal processes. Further development of this work will be in the direction of (a) increasing the precision of the thermal model for this particular application (b) develop a theoretical generic framework for the combined model and data-driven estimation approach to be used in other applications.

## Figures and Tables

**Table 1:** The parameters of the induction machine.

$R_{r}\left[\Omega \right]$ @ 20 °C	$R_{s}\left[\Omega \right]$ @ 20 °C	$L_{\mathrm{ls}}\left[H\right]$	$L_{\mathrm{lr}}\left[H\right]$	$L_{m}\left[H\right]$	Pole Pairs
0.0023	0.0024	$5.23\times 10^{-6}$	$5.23\times 10^{-6}$	$1.586\times 10^{-4}$	4
$f_{s}\left[Khz\right]$	$P_{n}\left[KW\right]$	$V_{n}\left[V\right]$	$T_{max}\left[Nm\right]$	$\eta_{max}\left[RPM\right]$	$\eta_{n}\left[RPM\right]$
200	6	48	60	18,000	4500

**Table 2:** Table of parameters for the observation model and particle filter.

**Model Coefficient**	**Model Coefficient**	**Filter Time Constant**
$\alpha_{1}$	$\alpha_{2}$	τ[s]
14.8052	1.3332	0.01
**Sample Time**	**Measurement Noise**	**Number of Particles**
h[s]	$R_{m}$[°C]	$N_{p}$
0.0015	4.3641	60

**Table 3:** Performance metrics for test scenarios 1–3.

Test Scenario	MSE	MAE	MAX	VAF
***I***	3.42	1.30 °C	6.90 °C	98.15%
***II***	2.86	1.16 °C	6.31 °C	98.52%
***III***	3.80	1.79 °C	7.21 °C	97.56%

**Table 4:** Table of weights and biases of the NARX network for 100 epochs.

$W_{l1,1}$	$W_{l1,2}$	$W_{l1,3}$	$W_{l1,4}$	$W_{l1,5}$	$W_{l1,6}$	$W_{l1,7}$	$W_{l1,8}$	$W_{l1,9}$	$W_{l1,10}$
0.1403	1.2166	0.1194	−1.4536	−1.1580	−1.4596	−0.0907	0.9794	0.0861	1.1758
$W_{l2,1}$	$W_{l2,2}$	$W_{l2,3}$	$W_{l2,4}$	$W_{l2,5}$	$W_{l2,6}$	$W_{l2,7}$	$W_{l2,8}$	$W_{l2,9}$	$W_{l2,10}$
2.0016	−0.5212	1.8543	−1.4572	−0.1994	−1.4414	0.0046	−1.7634	−0.6388	−0.4281
$W_{l3,1}$	$W_{l3,2}$	$W_{l3,3}$	$W_{l3,4}$	$W_{l3,5}$	$W_{l3,6}$	$W_{l3,7}$	$W_{l3,8}$	$W_{l3,9}$	$W_{l3,10}$
0.5346	−1.2230	−0.8087	0.1372	−1.4326	1.0812	−1.6871	−0.6863	0.7085	0.3366
$W_{l4,1}$	$W_{l4,2}$	$W_{l4,3}$	$W_{l4,4}$	$W_{l4,5}$	$W_{l4,6}$	$W_{l4,7}$	$W_{l4,8}$	$W_{l4,9}$	$W_{l4,10}$
−1.9551	0.7925	−1.0801	0.6204	−0.3679	−0.2235	−0.5951	0.8561	2.0562	1.2913
$W_{D1}$	$W_{D2}$	$W_{D3}$	$W_{D4}$	$W_{D5}$	$W_{D6}$	$W_{D7}$	$W_{D8}$	$W_{D9}$	$W_{D10}$
0.8833	0.0223	1.0860	−2.0130	−0.1146	−0.9997	1.4808	−0.3406	−1.8001	1.6212
$W_{L1}$	$W_{L2}$	$W_{L3}$	$W_{L4}$	$W_{L5}$	$W_{L6}$	$W_{L7}$	$W_{L8}$	$W_{L9}$	$W_{L10}$
−0.6887	−0.4060	−0.3728	0.4766	−0.7304	−0.6666	0.5783	−0.5917	0.7903	−0.7827
$b_{L1}$	$b_{L2}$	$b_{L3}$	$b_{L4}$	$b_{L5}$	$b_{L6}$	$b_{L7}$	$b_{L8}$	$b_{L9}$	$b_{L10}$
2.0631	−2.0804	−0.7474	0.1600	−0.5823	−0.0132	−0.2237	−0.4601	1.7474	2.1912
$b_{o}$	-	-	-	-	-	-	-	-	-
−0.0661	-	-	-	-	-	-	-	-	-

## Data Availability

Data sharing is not applicable to this article.

## Abbreviations

The following abbreviations are used in this manuscript:

BDB	Belt-Driven Booster
CAN	Controller Area Network
ECU	Electronic Control Unit
HEV	Hybrid Electrical Vehicle
IM	Induction Machine
LM	Levenberg-Marquardt
Mild-HEVs	Mild-hybrid Electrical Vehicles
NARX	Nonlinear AutoRegressive network with eXogenous inputs
PDF	Probability Density Function
PF	Particle Filter
PMSM	Permanent Magnet Synchronous Machine

## List of Symbols

The following nomenclature is used throughout the manuscript:

$V_{ds}$, $V_{qs}$	[V] voltage equilibrium of the stator in rotating frame;
$V_{dr}$, $V_{qr}$	[V] voltage equilibrium of the rotor in rotating frame;
ω	[rad/s] mechanical angular velocity;
$\omega_{r}$	[rad/s] electrical angular velocity;
$i_{ds}$, $i_{qs}$	stator currents in rotating frame;
$i_{dr}$, $i_{qr}$	[A] rotor currents in rotating frame;
$\psi_{qs}$, $\psi_{ds}$	[Wb] stator fluxes in rotating frame;
$\psi_{qr}$, $\psi_{dr}$	[Wb] rotor fluxes in rotating frame;
$L_{s}$	[H] inductance of the stator;
$L_{r}$	[H] inductance of the rotor;
$L_{m}$	[H] mutual inductance;
$L_{ls}$	[H] leakage inductance of the stator;
$L_{lr}$	[H] leakage inductance of the rotor;
$i_{r}$	[A] estimated value of the magnetization current;
$i_{s}$	[A] stator current;
$\tau_{r}$	[s] time constant of the rotor electrical dynamics;
$f_{s}$	[Hz] inverter switching frequency;
$R_{r}$	[Ω] nominal rotor resistance;
$R_{s}$	[Ω] nominal stator resistance;
$R_{m},R_{net}$	normal distribution variances of observation and transition model;
τ	[s] time constant of the low-pass filter introduced for realizability;
$\alpha_{1},\alpha_{2}$	coefficients of the thermal model;
$T_{s}$	[${}^{\circ }$C] stator temperature;
$T_{r}$	[${}^{\circ }$C] rotor temperature;
$T_{\alpha }$	[${}^{\circ }$C] predicted temperature with the thermal model;
$T_{net}$	[${}^{\circ }$C] predicted temperature with the NARX model;
${\hat{T}}_{r}$	[${}^{\circ }$C] posterior-estimated value of the rotor temperature;
*h*	[s] sample time;
η	[RPM] mechanical speed of the rotor measured in *revolutions per minute*;
Δ	gradient;
∗	reference value;
*w*	[-] particle filter sample weights;
$f_{1}$	sigmoid activation function of the hidden layer;
$f_{2}$	linear activation function of the output layer;
